# IDEAL: Maintaining PHC-focused training in a MBChB programme through a COVID-induced innovation

**DOI:** 10.4102/phcfm.v16i1.4389

**Published:** 2024-03-11

**Authors:** Ian Couper, Julia Blitz, Therese Fish

**Affiliations:** 1 Ukwanda Centre for Rural Health, Faculty of Medicine and Health Sciences, Stellenbosch University, Cape Town, South Africa; 2 Centre for Health Professions Education, Faculty of Medicine and Health Sciences, Stellenbosch University, Cape Town, South Africa; 3 Department of Clinical Services and Social Impact, Faculty of Medicine and Health Sciences, Stellenbosch University, Cape Town, South Africa

**Keywords:** medical education, learning, undergraduate, PHC, innovation, curriculum, co-creation, COVID-19

## Abstract

Responding to the need for authentic clinical training for students in the context of coronavirus disease 2019 (COVID-19), the Stellenbosch University Faculty of Medicine and Health Sciences developed an innovative 12-week longitudinal, integrated rotation for pre-final-year medical students, the Integrated Distributed Engagement to Advance Learning (IDEAL) rotation. This saw 252 students being placed across 30 primary and secondary healthcare facilities in the Western and Northern Cape provinces. With a focus on service learning, the rotation was built on experiences and research of members of the planning team, as well as partnership relationships developed over an extended period. The focus of student learning was on clinical reasoning through being exposed to undifferentiated patient encounters and the development of practical clinical skills. Students on the distributed platform were supported by clinicians on site, alongside whom they worked, and by a set of online supports, in the form of resources placed on the learning management systems, learning facilitators to whom patient studies were submitted and wellness supporters. Important innovations of the rotation included extensive distribution of clinical training, responsiveness to health service need, co-creation of the module with students, the roles of learning facilitators and wellness supporters, the use of mobile apps and the integration of previously siloed learning outcomes. The IDEAL rotation was seen to be so beneficial as a learning experience that it has been incorporated into the medical degree on an ongoing basis.

**Contribution:**

Longitudinal exposure of students to undifferentiated patients in a primary health care context allows for integrated, self-regulated learning. This provides excellent opportunities for medical students, with support, to develop both clinical reasoning and practical skills.

## Introduction

At the start of the coronavirus disease 2019 (COVID-19) pandemic (2020), clinical services at Tygerberg Hospital, the main teaching centre linked to the Faculty of Medicine and Health Sciences (FMHS) of Stellenbosch University (SU), were reorganised in response to the new patient profile and load. Clinical staff were redeployed into areas beyond their usual discipline. To assist this transition and in compliance with national disaster regulations, the FMHS temporarily withdrew all students from the clinical platform. Within 3 months, Tygerberg Hospital was able to accommodate students again, but in lower numbers. The final-year medical class was prioritised, leaving the challenge of how pre-final-year medical students could recommence clinical training so as to graduate on time at the end of 2021. In response to this challenge, the Integrated Distributed Engagement to Advance Learning (IDEAL) rotation was designed for this cohort.

The IDEAL rotation was introduced as a 12-week integrated rotation with two overarching objectives: (1) to offer a learning opportunity that would enable students to integrate elements of most clinical disciplines through patient encounters, based on a primary care philosophy and (2) to assist in providing service in the health system through students joining healthcare teams. This built on our existing opportunities for students to be placed on the distributed platform in the Western and Northern Cape, in sites that included regional and district hospitals, community health centres and clinics. We understood that the historical format of discipline-based rotations would not be feasible at most of these sites, as specialist supervision was not available at that time.

## Development and implementation of the IDEAL rotation

The decision to implement this rotation, while catalysed by the pandemic, arose from discussions with student representatives and drew on our experience over many years of placing students at distributed sites, whether rural, peri-urban or urban. The design team consisted of faculty management, academic staff from a range of disciplines, Bachelor of Medicine and Surgery (MB ChB) unit representatives, the Rural Clinical School (RCS), business management, placement logistics team and student representatives. In designing an academic programme to fit the needs, we drew together numerous strands. As IDEAL replaced an existing 4-week primary healthcare rotation, this formed the basis of the design. To accommodate the other major specialty rotations in the final year, which had to be shortened, some of their final-year outcomes that were achievable at the primary or secondary care level were included in IDEAL. The choices were informed by experiences of various members of the team, for example, in the Integrated Primary Care rotation at the University of the Witwatersrand^1,2^; the Expert Reference Group planning for the new medical programme at Nelson Mandela University; the development of the SU RCS, which had been established in 2011.^3^ The RCS provides an option for students to spend their entire final year at a district hospital site,^4^ offering students a fully integrated clinical experience.^5^ Importantly though, our decisions were also informed by research that members of the team had been involved in over the preceding years – a national initiative focused on distributed clinical training, which ultimately led to the development of a framework,^6^ as well as research looking at the impact of placing students in health facilities.^7,8^

## Student learning

Clinical staff at the distributed training sites were also responding to the COVID pandemic; thus an important component of the design was for the fifth-year students to join teams and contribute to service delivery at the sites. Staff were not asked to teach the students in any format other than patient-based (‘bedside’) training, nor to assess the students.

A significant shift was required of students to develop more self-regulated learning habits and to become integral members of clinical teams, taking partial responsibility for patient care and their own learning. The rotation was structured as alternating days with the first being on the clinical platform and the next being a learning day during which students could complete their learning tasks and assignments.

Three support measures were put in place. Firstly, the students’ learning management system (SUNLearn) provided a space for online resources and for students to post queries for relevant specialists to respond asynchronously. Secondly, doctors in the faculty volunteered to serve as learning facilitators (LFs). The students logged patient encounters on a mobile app (VulaMobile) using a modified SNAPPS template,^9^ which the LFs then used to assist the students to explore the clinical reasoning used in the patient encounter. This did not require discipline-specific knowledge, as the objective was to facilitate the learning of clinical reasoning. Thirdly, volunteer wellness supporters from a range of different professional backgrounds were appointed to check in with students, basing their conversations on short reflective pieces submitted online weekly by students. These supporters served as a sounding board for students as they encountered new clinical experiences and ways of learning.

## Partnership

Existing partnerships with the two provincial health departments formed the basis for engagement with the training sites for IDEAL. Prior to the pandemic, the FMHS had over 100 sites as part of the distributed training platform for undergraduate programmes. The IDEAL rotation used many of these and several new sites to expand capacity. A total of 252 fifth-year medical students were placed in 12 healthcare complexes, consisting of stand-alone facilities such as district hospitals or regional hospitals with their primary care facilities, comprising 30 facilities in total.

## Innovations

The IDEAL rotation included the following significant innovations:

- **Extensive distribution of clinical training**: For the first time in South Africa, an entire, large medical school class was distributed across an extended training platform at the same time and for so long.
- **Responsiveness to health service needs:** Early consultation enabled the team to build health departments’ COVID-19 requirements into the academic planning.
- **Co-creation of the module with the involved students:** Students not only were the ones to raise the possibility of such a programme, but they were also fully part of the development and implementation team. Class representatives were very active, constantly consulting their peers and providing bidirectional feedback.
- **Role of learning facilitators:** Forty-five faculty members from all disciplines were recruited as LFs. Each was allocated between four and six students, who submitted five patient presentations every fortnight. Learning facilitators responded with questions and suggestions to facilitate patient-centred learning and met online with students individually or in groups to discuss learning issues every 2 weeks. For many of these faculty members, this precipitated a move from teaching to facilitation of learning.
- **Use of mobile apps:** Students were asked to submit a patient encounter on every clinical day via VulaMobile, a referral app used across levels of care.^10^ The app allowed the leadership team to monitor the number of patients submitted per student and the responsiveness of LFs. Procedures performed were logged on a purpose-built Microsoft Power App, My Clinical Logbook, which listed 90 procedures across all disciplines, indicating expected level of competence and suggested numbers for each procedure. Regular analysis of procedures per student per site and the frequency of procedures done allowed rapid identification of underperforming students who could then be supported. During the rotation, students asked for extra procedures to be listed, another example of co-creation.
- **Wellness supporters:** Twenty-five faculty volunteers assisted in supporting 10 students each. Students submitted a weekly journal about their learning, in a variety of formats. This provided psychosocial support to students who were away from home at a difficult time; student participation was voluntary and not part of assessment. Referrals to student support services were suggested where indicated.
- **Integration of previously siloed learning outcomes:** Common outcomes were set regardless of site and exposure, focused on patient encounters and the final assessment took the form of an integrated practical exam. On-site supervision by local clinicians was mostly provided by generalists, particularly family physicians.

## Conclusions

Reflecting on our own learning journey, we realised that it was remarkable what we had achieved in such a short time. This was only possible because we all shared the same goals, we grounded our approach strongly in learning theory, we had a substantial basis of experience and evidence and we built on existing relationships within the faculty and with our service partners. It required significant risk taking, which the context of COVID-19 facilitated. The process embodied the responsive adaptability we had identified as a key enabler for distributed training.^6^

The IDEAL rotation exemplifies authentic learning in the real-world clinical context, focused on primary healthcare. The positive experience of students and the extent of learning that occurred led to the ongoing inclusion of the IDEAL rotation in the MBChB curriculum, supported by final-year specialist disciplines that noticed the difference in the confidence and participation of students built during the prior IDEAL experience. Adaptations have been made over the years, but the key principles remain intact. The IDEAL rotation foreshadows and offers lessons for a module planned as part of the renewed SU MBChB curriculum, which intends to distribute all medical students outside of the central academic hospital for their final 36 weeks.
